# Diversifying the genomic data science research community

**DOI:** 10.1101/gr.276496.121

**Published:** 2022-07

**Authors:** The Genomic Data Science Community Network

**Affiliations:** 2 Clovis Community College, Fresno, CA 93730, USA;; 3 Biology Department, El Paso Community College, El Paso, TX 79924, USA;; 4 US Fish and Wildlife (Northwest Indian College), Onalaska, WI 54650, USA;; 5 Biology Department, Spelman College, Atlanta, GA 30314, USA;; 6 Turtle Mountain Community College, Belcourt, ND 58316, USA;; 7 Department of Biological Sciences, University of Southern California, Los Angeles, CA 90089, USA;; 8 Biology Department, Claflin University, Orangeburg, SC 29115, USA;; 9 Department of Biostatistics, Johns Hopkins Bloomberg School of Public Health, Baltimore, MD 21205, USA;; 10 National Human Genome Research Institute, National Institutes of Health, Bethesda, MD 20892, USA;; 11 Department of Quantitative Health Sciences, University of Hawaii at Manoa, Honolulu, HI 96822, USA;; 12 Smithsonian Institute National Museum of Natural History, Washington, DC 20560, USA;; 13 Guttman Community College, New York, NY 10018, USA;; 14 Department of Microbiology and Biomedical Sciences, Dine College, Tuba City, AZ 86045, USA;; 15 Fred Hutchinson Cancer Research Center, Seattle, WA 98109, USA;; 16 Department of Biology, Johns Hopkins University, Baltimore, MD 21218, USA;; 17 Department of Biology, Northern Virginia Community College–Alexandria, Alexandria, VA 22311, USA;; 18 Department of Chemistry and Biochemistry, Fort Lewis College, Durango, CO 81301, USA;; 19 Department of Neurobiology, Morehouse School of Medicine, Atlanta, GA 30310, USA;; 20 Science and Technology, Universidad Ana G. Méndez–Carolina, Carolina, 00983, Puerto Rico;; 21 Natural Sciences Department, University of Puerto Rico at Aguadilla, Aguadilla, 00603, Puerto Rico;; 22 Department of Molecular and Cell Biology, University of California, Merced, Merced, CA 95343, USA;; 23 School of Graduate Studies and Research, Meharry Medical College, Nashville, TN 37208, USA;; 24 Department of Biological Sciences and Border Biomedical Research Center, University of Texas at El Paso, El Paso, TX 79968, USA;; 25 Department of Math, Statistics, and Data Science, Montgomery College, Rockville, MD 20850, USA;; 26 Departments of Biology and Computer Science, Johns Hopkins University, Baltimore, MD 21218, USA;; 27 Chemical and Biological Sciences, Montgomery College, Germantown, MD 20876, USA;; 28 Department of Biology, University of Puerto Rico–Ponce, Ponce, 00732, Puerto Rico;; 29 Department of Embryology, Carnegie Institution, Baltimore, MD 21218, USA;; 30 National Institutes of Health, Bethesda, MD 20892, USA;; 31 Department of Biology, Flathead Valley Community College, Kalispell, MT 59901, USA;; 32 Department of Biology, Nevada State College, Henderson, NV 89002, USA;; 33 Department of Biology, Virginia State University, Petersburg, VA 23806, USA;

## Abstract

Over the past 20 years, the explosion of genomic data collection and the cloud computing revolution have made computational and data science research accessible to anyone with a web browser and an internet connection. However, students at institutions with limited resources have received relatively little exposure to curricula or professional development opportunities that lead to careers in genomic data science. To broaden participation in genomics research, the scientific community needs to support these programs in local education and research at underserved institutions (UIs). These include community colleges, historically Black colleges and universities, Hispanic-serving institutions, and tribal colleges and universities that support ethnically, racially, and socioeconomically underrepresented students in the United States. We have formed the Genomic Data Science Community Network to support students, faculty, and their networks to identify opportunities and broaden access to genomic data science. These opportunities include expanding access to infrastructure and data, providing UI faculty development opportunities, strengthening collaborations among faculty, recognizing UI teaching and research excellence, fostering student awareness, developing modular and open-source resources, expanding course-based undergraduate research experiences (CUREs), building curriculum, supporting student professional development and research, and removing financial barriers through funding programs and collaborator support.

## Foundations for justice in genomic data science

Despite growing opportunities in data science careers, systemic barriers have limited the participation of underrepresented groups in genomic data science research and education (Canner et al. 2017). Among bachelor's degree recipients in biological sciences, computer sciences, mathematics, and statistics from 2006–2016, 8.7% were Hispanic or Latinx, 7.8% were Black or African American, and 1.9% were multiracial and/or indigenous American (National Science Foundation 2019a). Meanwhile, these groups represent 16.3%, 12.3%, and 2.5% of the US resident population, respectively (National Science Foundation 2019a). Disparities are more pronounced in graduate education (Wiley et al. 2020). Affinity organizations in which members of underrepresented groups come together are vital to developing a sense of belonging and support system (Supplemental Table S1). However, for true representation in research, science needs inclusive spaces where researchers can communicate actively with educators and where students are supported in developing science, technology, engineering, and mathematics (STEM) identities.

The technological advancements of high-throughput sequencing in the past two decades have enabled the rapid proliferation of genomic data (Goodwin et al. 2016) but they have also led to an even greater access imbalance. Over 60 petabases of data (National Center for Biotechnology Information 2021), or about a million times the size of the original human genome project (International Human Genome Sequencing Consortium 2001), is currently available within the US National Center for Biotechnology Information (NCBI) genomic sequencing repositories. This wealth of data will help scientists determine disease risk, diagnose rare conditions, improve drug safety and efficacy (Manolio et al. 2019), survey pathogens for public health applications (Khoury et al. 2020), and even combat the effects of climate change (Hoffmann et al. 2021). Our greatest limitation is personnel to interpret these data. Yet, genomic data science currently lacks a scaffolded mechanism that supports all individuals and provides a hub of intellectual capital, curated genomic data, and the infrastructure required for authentic learning gained through research experiences. Broader, more diverse participation should be the starting point for creating a more inclusive genomic data science field (Mapes et al. 2020). Focusing on participation is not only ethical but desirable for more novel solutions to problems (Hofstra et al. 2020) and is necessary for bringing different perspectives to the table (Zook et al. 2017).

**Figure 1.**
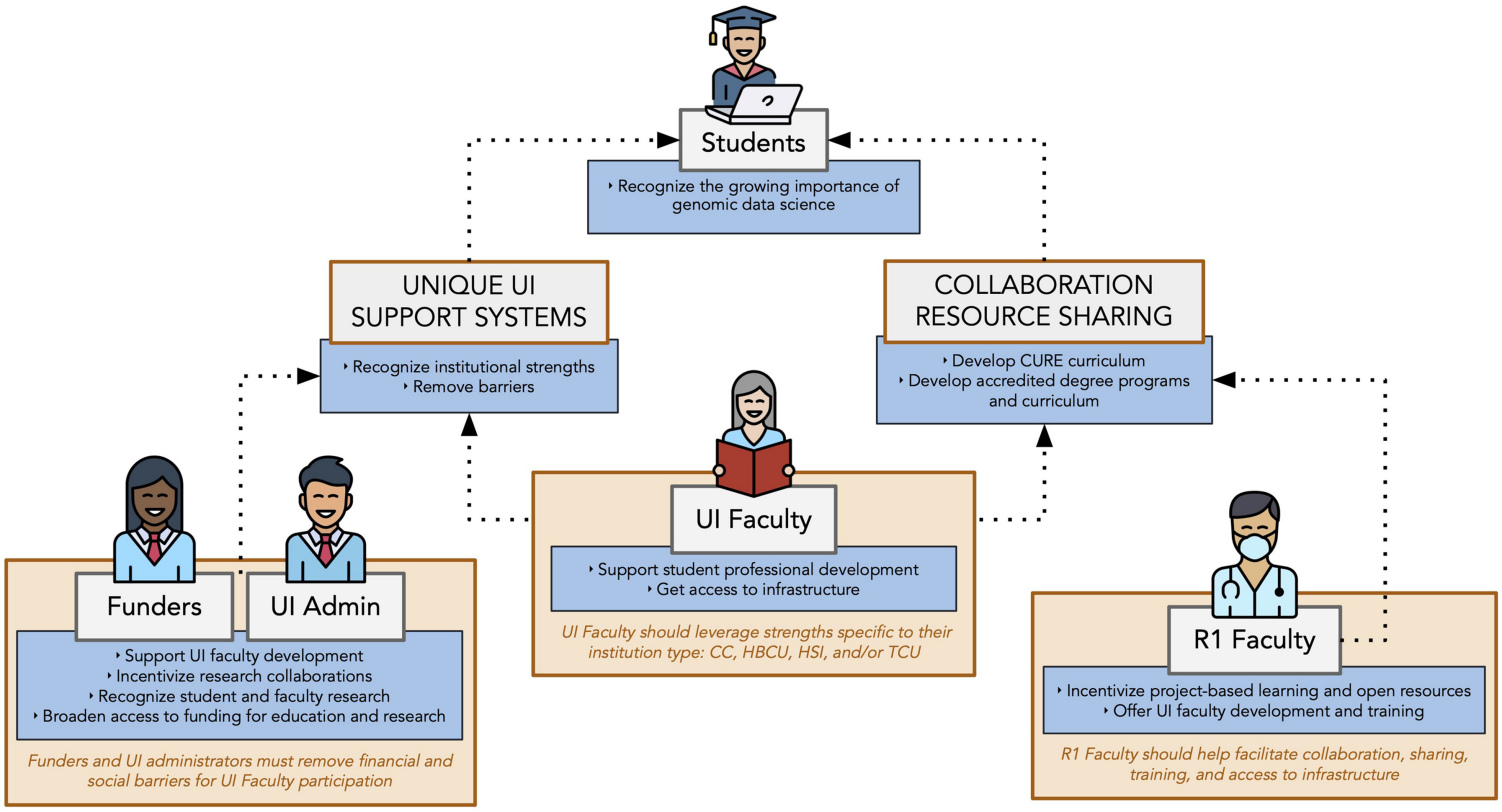
Our vision for diverse genomic data science. The Genomic Data Science Community Network strives for a just and fair system in which researchers, educators, and students are engaged in genomic data science research regardless of their institution's scientific clout, resources, geographical location, or infrastructure. We propose specific actions (dark/blue boxes) that can be taken by research and educational community stakeholders (light/gray boxes). Support mechanisms are also outlined (italic/orange boxes/hashed lines). (UI) Underserved institution, (CC) community college, (HBCU) historically Black college or university, (HSI) Hispanic-serving institution, (TCU) tribal college or university.

Our vision for a diverse scientific community engaged in genomic data science research is one in which researchers, educators, and students thrive in a just and fair system, not limited by their institution's scientific clout, resources, geographical location, or infrastructure (Fig. 1). Here, we focus on traditionally underserved institutions (UIs) in the United States, which include minority serving institutions (MSIs) defined by the US Department of Education: historically Black colleges and universities (HBCUs), Hispanic-serving institutions (HSIs), and tribal colleges and universities (TCUs) (Li and Carroll 2007). UIs also include community colleges (CCs) and some primarily undergraduate institutions that overlap substantially with MSIs (Nguyen et al. 2015). Collectively, UIs play a critical role in educating ethnically, racially, and socioeconomically underrepresented students despite limited access to resources (Li and Carroll 2007). In addition to the number of traditionally underrepresented students educated at UIs, these colleges and universities possess unique strengths, such as the greater sense of belonging, more positive mentoring relationships, and role modeling (Gin et al. 2021; Jayabalan et al. 2021). Faculty and staff at these institutions also have experience with the specific interests, needs, challenges, and concerns of the populations they serve (Text S1). UIs are therefore essential to removing systemic bottlenecks that lead to a homogenous workforce.

**Figure 2.**
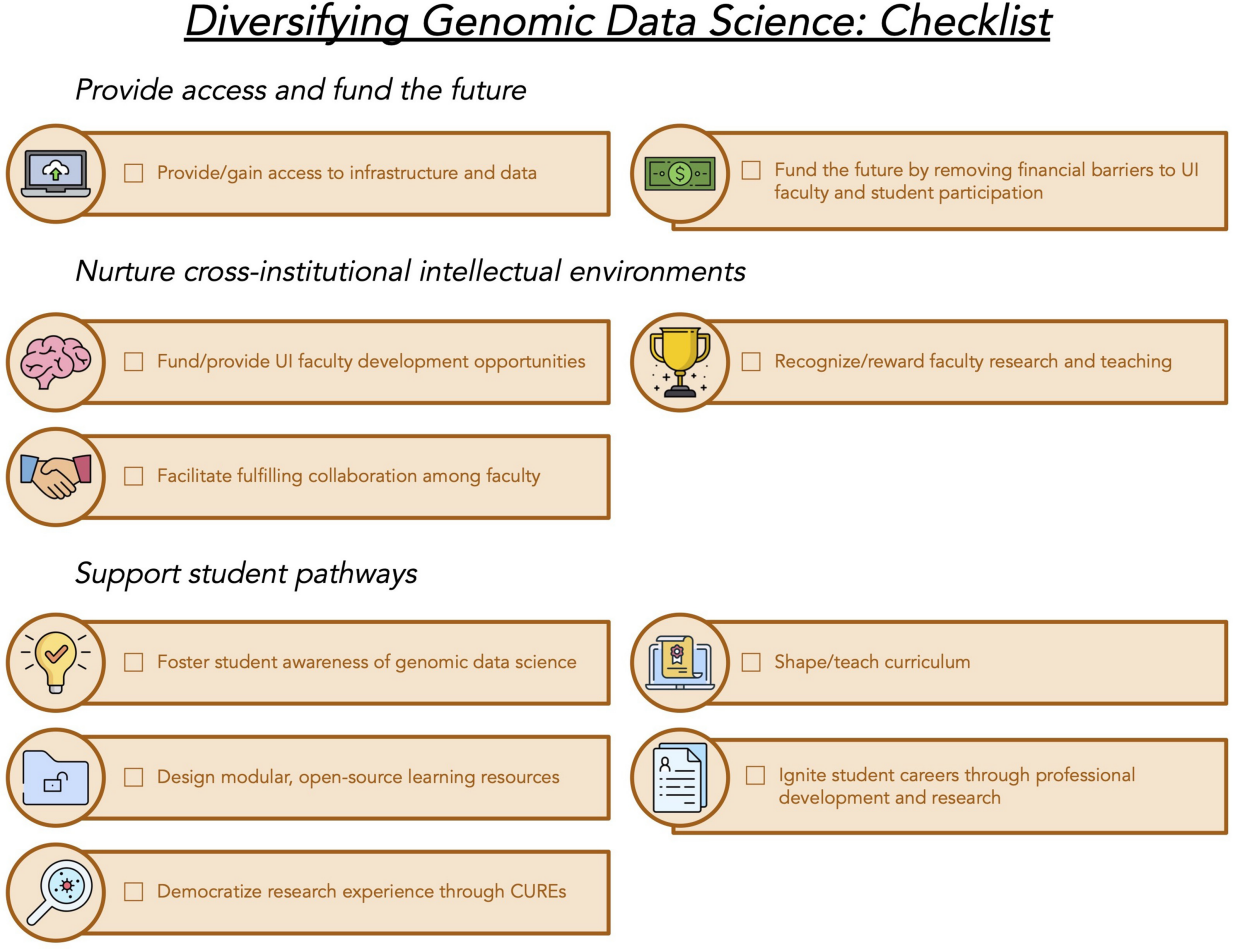
Checklist of actions for diverse genomic data science. We envision greater participation in genomic data science with these key actions. (UI) Underserved institution; (CURE) course-based undergraduate research experiences.

In this perspective, we share lessons learned from forming the institutionally diverse Genomic Data Science Community Network (GDSCN; http://www.gdscn.org/). Throughout a series of symposiums, one-on-one meetings, and electronic communications, we have provided a platform to listen to the needs of faculty constituents from UIs. Together, we have identified the needs and opportunities in our current academic sphere and its limitations to achieving better representation. Although there are several outstanding communities and recent insights for broadening participation in bioinformatics (Buchwald and Dick 2011; Garrison et al. 2019; Hemming et al. 2019; Jayabalan et al. 2021), here we focus on challenges shared broadly by faculty at UIs and action items that can be taken to address them (Fig. 2). We highlight ways in which outside funders and researchers at R1 institutions—doctoral universities with very high research activity (McCormick and Zhao 2005)—can support their colleagues at UIs. With support from funding institutions and a dedicated GDSCN membership, we believe our model will contribute to the organic diversification of genomic data science research.

## Provide access to infrastructure and data

One of the most immediate inequalities is access to data storage and computing power, which are essential prerequisites for genomic data science analyses. Financing computing clusters typically owned and managed by R1 institutions is cost limiting for most UIs, especially if a limited number of faculty members will use it. Cloud computing resources are becoming an increasingly attractive alternative. Institutions only pay for the computing time used, have a lower barrier to entry, require no upfront investment, and ensure students have equal access to software versions (Supplemental Table S2; Navale and Bourne 2018). For example, the AnVIL platform (Schatz et al. 2022) provides multiple entry points for different levels of experience, from command line access and R/Python notebooks to the no code–needed Galaxy user interface (Jalili et al. 2020). Fees for compute time and disk storage space are explicitly stated upfront. Other examples include the CyVerse Discovery Environment (Goff et al. 2011; Merchant et al. 2016), which provides a cloud computing space and includes tools for teaching, as well as Galaxy's cloud platform (https://usegalaxy.org/). Laptop or device carts for laboratories or projects can also reduce the cost burden on students. Combined with affordable devices, such as Chromebooks or tablets, cloud computing can be a powerful, low-barrier resource for democratizing authentic data science experiences in general.

## Nurture cross-institutional intellectual environments

### Fund ongoing faculty development

Broadening participation in genomic data science research depends on a strong support system for faculty at UIs. Yet, resources at these institutions are often stretched thin, making hiring and training the appropriate personnel extremely difficult. Many faculty also have high teaching loads with little time for continuing education, leaving instructors underprepared to teach rapidly evolving genomic data science topics (Williams et al. 2019; Zhan et al. 2019). Financing and incentivizing professional development outreach programs can help meet these challenges. Organizations can follow other successful programs, such as the National Human Genome Research Institute's (NHGRI) Short Course in Genomics (Robbins et al. 2021). This program provides lectures and hands-on learning and has successfully reached over 100 faculty that continue to teach in diverse communities. Events are also hosted by the IDeA Networks of Biomedical Research Excellence (INBRE) (National Institutes of Health 2021b). Continued mentorship and resource sharing from NIH researchers and staff after completion of the short course also ensure longer-term positive outcomes.

Outside of government-sponsored programs, small grants funding workshops, conferences, or assistants (such as research or teaching assistants) can substantially empower UI faculty. Researchers at R1 institutions can build on-demand materials for self-directed faculty education and/or tailor “train the trainer” workshops for faculty needs and interests. For example, the Jackson Laboratory designed a week-long course, Big Genomic Data Skills Training for Professors, to train small college and regional university faculty (Zhan et al. 2019). Leaders at R1 institutions should commit to honoring these contributions when considering teaching, service, outreach, and/or tenure committee decisions.

Within UIs, administrators should encourage protected time and teaching release for attending workshops, writing proposals, and/or developing new curriculum. They should incentivize faculty members to pursue educational and research opportunities in genomic data science for the students and the university. Dedicated sabbatical time allows UI faculty to develop research and coursework with outside faculty (Hemming et al. 2019) and innovate new student experiences (Yarmohammadian et al. 2018). Future initiatives can capitalize on a sabbatical internship/exchange model, in which faculty can choose to travel to different research laboratories and gain exposure to additional research initiatives and peer mentorship. Not only do these professional experiences enhance skill-building, but also they help future collaborators find one another and organically build collegiate networks.

Faculty members at UIs who have research expertise should be incentivized to build up their colleagues through peer sharing and learning. Programs like the Centers of Research Excellence in Science and Technology (CREST) and HBCU Research Infrastructure for Science and Engineering (HBCU-RISE) (National Science Foundation 2017a), as well as INBRE, specifically support these efforts within UIs. Additionally, the recently established NHGRI Office of Training, Diversity, and Health Equity (TiDHE) aims to coordinate training and career development for a more diverse genomics workforce (National Institutes of Health 2021c). Faculty who are entirely dedicated to teaching can use these funds to engage students in authentic research activities and to explore pedagogical studies. Furthermore, with administrative support within the same institution, experienced faculty can help develop “pathways” (from beginner to more advanced) by which their colleagues can progressively learn (Crown et al. 2011). Going beyond one-off workshop experiences to form development plans could help make faculty participation both effective and scalable.

### Facilitate fulfilling collaboration

Collaboration among institutions ensures faculty come in contact with a diversity of topics and perspectives and are set up for success, from proposal to publication (Hemming et al. 2019). All UI faculty should consider meeting collaborators through established programs like the National Research Mentoring Network (NRMN) (Hemming et al. 2019; https://nrmnet.net/), which connects experienced research mentors with student and faculty mentees from MSIs. The Oak Ridge Institute for Science and Education (ORISE) matches faculty with mentors for hands-on research experience at one of the U.S. Department of Energy's sponsoring agencies (https://orise.orau.gov/). Affinity groups, such as the Native Investigator Development Program (NIDP) and others (Supplemental Table S1), can also contribute toward fruitful, collaborative relationships (Buchwald and Dick 2011). Government programs can also help sponsor additional community building. For example, the Research Coordination Networks Program aims to foster synthesis, new collaborations, and resource sharing while advancing research and education (National Science Foundation 2017b).

Collaborative networks must position UI faculty in leadership roles while actively engaging them in setting goals and priorities. Currently, only a small number of programs specifically target these faculty. For example, the INBRE and Bridges to the Baccalaureate (National Institutes of Health 2021a) programs sponsor collaboration between two-year and four-year UI teaching institutions and R1 research institutions with the goal of supporting faculty and providing student research opportunities. Other collaborative networks, such as the Network for Integrating Bioinformatics into Life Sciences Education (NIBLSE), aim to support faculty in bioinformatics coursework while encouraging professional achievement via publication (Dinsdale et al. 2015). In addition to connecting through scientific conferences and meetings, hands-on workshops for faculty and/or students, bioinformatics outreach efforts, and seminar series, these programs can help move toward balancing access to collaborative networks.

### Recognize faculty research and teaching

Recognizing the research breakthroughs by faculty has a twofold benefit: It rewards faculty members’ hard work and raises awareness and discussion in the broader community. Sharing the research experiences on social media platforms, such as Twitter (https://twitter.com/), is perhaps the fastest and easiest way to gain academic recognition and raise awareness (Cheplygina et al. 2020). Faculty should work together to amplify each other's work. Social media–savvy faculty might consider social media workshops for those who are not used to engaging on these platforms. Where possible, faculty should leverage their institutional press/media office to create releases and/or posts or use organizations that aggregate blog or social media posts. Using social media and blogs to disseminate work can raise important questions, such as when and how work should be shared among stakeholders, from public relations offices at institutions to tribal leadership. Research symposia can also serve to showcase faculty participation and collaboration while making them feel included in the community. Existing programs, such as the NHGRI Short Course in Genomics (Robbins et al. 2021), provide faculty the opportunity to present work to NIH teams, as well as other conference communities. Virtual conferences, having evolved quickly during the COVID-19 pandemic (Jarvis et al. 2020), can provide a low-cost and accessible way to feature faculty and student research experiences.

Faculty need to be acknowledged for their excellence in research as well as their hard work teaching and applying for funding, regardless of award status. Nonfinancial awards at all levels, from institution and school or division to department, are relatively easy to establish for innovative contributions to curriculum content, teaching, research, service, outreach, and mentoring. Administrators can show faculty they are valued by considering such awards in tenure or promotion decisions. As courses in genomic data science are often cotaught by faculty members from different disciplines or with different areas of expertise, university administrators should also create straightforward methods to attribute teaching credits to each participating faculty member and count them in the member's total teaching load. Overall, empowering UI faculty will lead to greater diversity among faculty researchers in genomic data science (Whittaker et al. 2015). Improved faculty representation also paves the way for improved student achievement, recruitment, and retention (Merisotis and McCarthy 2005).

## Engineer multiple student pathways

### Foster student awareness

With infrastructure and a faculty foundation established, engaging students and the community directly is required for broadening long-term student participation. In fact, a recent survey highlights that a tribal community has specifically requested more genetics education within their community (Claw et al. 2021). Based on our assessment of the role of CCs, HBCUs, HSIs, and TCUs within their communities, these are the best institutions to provide this education. However, students starting their postsecondary education with interests in biological sciences might have little to no exposure to genomic data science as a potential area of study and career path. Moreover, the sheer volume of topics to cover in introductory biology courses and time needed for molecular “wet” laboratory instruction can make incorporating additional bioinformatics and genomics topics difficult. Existing resources also tend to be less specialized. Thus, to facilitate early student exposure to genomic data science, faculty likely need to restructure and/or update introductory biology courses to include essential material on genomics and bioinformatics.

To foster early awareness, instructors can briefly introduce genomic data science topics in first-year/freshman colloquia (e.g., Clark and Cundiff 2011), seminars, and professional/career development courses. Topics should highlight the challenging and fulfilling career opportunities and the potential for a meaningful impact within their communities. Technical development and bioethics should also be emphasized in these courses. Diverse interests, such as computer science, statistics, or other interdisciplinary majors, can be accommodated via course cross-listing. Departments can also host joint seminar series in which students have the opportunity to meet genomic data science researchers working in academic, industrial, and/or nonprofit careers. College administrators should support faculty as they develop best practices for introducing this rapidly growing discipline to students.

The final years of secondary school are a pivotal time for students. Faculty should focus on raising awareness, including via open houses, easy-to-find websites, workshops, and weekend learning experiences. Reaching out to high school student STEM clubs, or sponsoring the development of such clubs, can serve as a channel by which to encourage participation. Providing opportunities for college or university visits for students can improve student self-efficacy and give them the tools they need to select their postsecondary career path (Glessner et al. 2017; Boatright 2020). A 1-d congress that brings students from multiple high schools to a college or university could allow students to meet practicing scientists. Early college high schools (ECHS) and dual-enrollment programs, which allow students to simultaneously earn a high school diploma and an associate's degree, could provide a conduit for reaching students historically underrepresented in higher education (Texas Education Agency 2020; Virginia Community Colleges 2020). Development of strong partnerships between the high school independent districts and higher education institutions is key to the success of these initiatives. The DataTrail (https://www.datatrail.org/) program also provides a successful model by which high schoolers and GED earners can connect directly to faculty mentors and data science employment.

Raising awareness among even younger students in primary and secondary school is a recognized need (Morgan et al. 2016). Fundamental education in programming (e.g., via games, Lindberg et al. 2019) can lay the foundation for scientific analysis; the genomic data science community should work with teachers to provide age-appropriate activities. Access to computers is a consistent challenge, although smart devices like phones and tablets can be powerful tools when combined with cloud resources (Supplemental Table S2). Despite technological advancements, preschool and kindergarten teachers often feel unsupported and underprepared to teach STEM, contributing to underrepresentation that is persistent and pervasive (Fuller et al. 2021). Remedying resource and training inequities must be a funding priority as government agencies consider broader initiatives to introduce data science more prominently into primary and secondary school education.

### Design modular, open-source learning resources

Most life science instructors agree that bioinformatics knowledge and skills are essential to biology students (Wilson Sayres et al. 2018). One of the great successes of data science more broadly is the proliferation of open-source and open-access resources. Many freely available online resources have emerged with the growing interest in genomic data science, including the Coursera Genomic Data Science Specialization (https://www.coursera.org/specializations/genomic-data-science) and Bioinformatics Algorithms (https://www.bioinformaticsalgorithms.org/). Both of these courses have already reached hundreds of thousands of students. Although massive online open courses (MOOCs) are broadening access to education, additional support (such as community building and awareness, confidence building, faculty training) is needed for learners from underrepresented groups (Stich and Reeves 2017). UI faculty can serve as important bridges between MOOC material and UI students, but instructors still face many ongoing challenges. UI faculty must wade through the wealth of material to find content that is appropriate and interesting for their target audience or is specific to a particular application, topic, or technique. Finding activities that are modular and interchangeable given their existing course lesson plans takes time. In some cases, restrictive licensing for “remixing” and updating as needed is a barrier. Many educators, UI and R1 alike, also struggle to keep pace with constantly evolving approaches (Ryder et al. 2020).

Because many resources already exist to teach data science more generally, new development can focus on free interactive platforms (Kross et al. 2020) and/or open-source methods for updating and remixing content to make it more accessible (Savonen et al. 2022). In general, resources teaching introductory concepts, foundational statistics, and programming skills should continue to be made open source wherever possible (Supplemental Table S3). For more specific genomics content, R1 developers should work with UI faculty to develop relevant, approachable content that can be freely accessed and tailored as needed. These partnerships can be incentivized by administration and sponsoring agencies by including it in promotion and/or funding decisions. Another successful approach is to include instructor guides within materials, such as the teaching instructions included with the modular microbiome QIIME2 activity developed by UI faculty (for GitHub link, see Lee 2019).

### Democratize research experience through CUREs

Undergraduate research experiences can improve outcomes for historically underrepresented students in the sciences (Carpi et al. 2017; Krim et al. 2019). Course-based undergraduate research experiences (CUREs) bring students directly to authentic research with their peers in a classroom setting and are defined by use of scientific practices, discovery, relevant and/or important work, collaboration, and iteration (Auchincloss et al. 2014). Whereas government-focused programs (such as the NSF Research Experiences for Undergraduates [REU] program) traditionally offer limited laboratory space to a small number of students, CUREs expose a larger group of students in tandem (Elgin et al. 2021). CUREs break down student financial and personal barriers and can minimize faculty members’ unconscious bias (Bangera and Brownell 2014). Furthermore, flexible CUREs can help establish a mutually uplifting, safe, and positive mentoring environment while creating a peer community in which students can collaborate, share discoveries, and support one another (Kokotsaki et al. 2016). Indeed, a sense of peer and institutional belonging is a key metric for student feelings of inclusion and retention in UIs (Winkle-Wagner and McCoy 2018; Sweeder et al. 2021).

Though CUREs have many benefits (Elgin et al. 2021), UI faculty sometimes receive pushback for implementing CUREs or requesting research classroom hours within established curricula. One solution is creating a variety of CUREs with flexible length/timing (weeks or semester long) in mind. For example, some instructors might find it easier to incorporate “mini-CUREs” that require only part of the semester within an existing course. Faculty can also consider incorporating CUREs within a senior capstone course. It is essential that faculty collectively maintain a support system for sustainable CURE implementation (Lopatto et al. 2014; Elgin et al. 2021). Research faculty at R1 institutions can contribute to this support system by helping develop CUREs, making them more accessible, and/or providing “train the trainer” resources to UI faculty colleagues. The Genomics Education Partnership (https://thegep.org/) provides training and resources for faculty implementing CUREs. Programs like the Small World Initiative (a student education program focused on widespread antibiotics resistance, http://www.smallworldinitiative.org/) have trained faculty at over 300 schools to incorporate authentic research into their classrooms.

**Figure 3.**
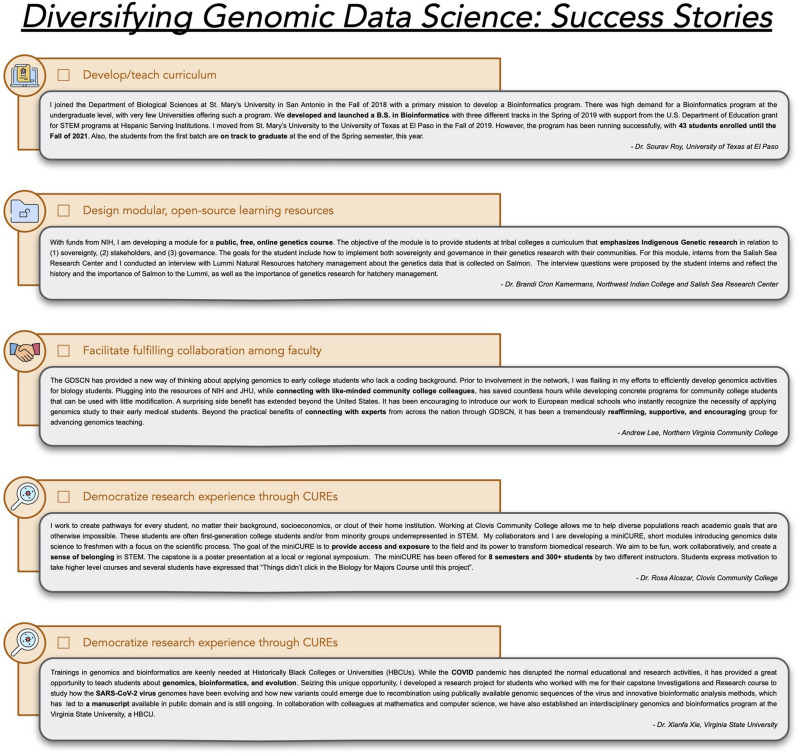
Success stories informing the right paths forward. GDSCN members provided nonexhaustive examples of successes at their institutions. These examples show how UI faculty can excel in nurturing collaborative environments and supporting student pathways with the right support network.

The key for success for any classroom content is to ensure it is relevant to real life and/or the students’ future career goals and that it is an authentic research experience for both the faculty and students involved. There are several examples of CUREs that have been created by UI faculty and/or implemented at UIs. The Science Education Alliance-Phage Hunters Advancing Genomics and Evolutionary Science (SEA-PHAGES; https://seaphages.org/) network is both an inclusive research education community and the developer of the two-term laboratory CURE studying viral diversity, evolution, genome annotation, and bioinformatic analyses (Jordan et al. 2014; Hanauer et al. 2017). Data generated by SEA-PHAGES students have been used in real-time medicinal interventions and high-impact publications (Dedrick et al. 2019), which can leave a lasting impact on students. The Clovis Community College Biol-12 Research in Biology course (https://www.cloviscollege.edu/landing/biol-12-genomics-data-science.html) introduces data science with a unique emphasis on university transferability while having no prerequisites (Fig. 3). This course also includes professional development for students, including presenting at a symposium and working on college or job applications. With funding from the NIH Bridges to the Baccalaureate Program, faculty at El Paso Community College developed introductory biology CURE projects focused on topics relevant to the local US–Mexico border community, including water contamination and antibiotic-resistant microorganisms. Faculty at Virginia State University designed a research program focused on SARS-CoV-2 for the required one-semester capstone course (Fig. 3). This work showed the real scientific impact of students and faculty to help reveal a biological process that has been largely overlooked by existing studies of the SARS-CoV-2 virus (Xie et al. 2021). Development of new CURE topics, for example, DNA forensics, population genetics of communities, or bioinformatics in nature, should thoughtfully engage with students throughout the design process.

### Shape curriculum

Accreditations for students participating in genomic data science courses and CUREs are critical for state-of-the-art education, retention, and enrollment growth. Yet, building new degree programs is challenging, especially for broad, rapidly evolving domains like genomic data science. Course names like “big data” might mean very different content, activities, platforms, and learning objectives among institutions. First, programs should use a “pathway model” consisting of clear program outlines/degree plans with distinct course sequences, learning objectives, progress milestones, and learning outcomes. Setting up guidelines and learning objectives that would need to be covered early on will also help guide individual courses. Standardizing learning outcomes and course titles (e.g., topic + tool, “genomic data science with Galaxy”) will provide a more detailed picture of the student's exposure to specific skills and topics. To allow students to explore tools more in depth, some institutions might find it beneficial to include coding languages (e.g., R or Python) as part of the computer literacy or general education requirements.

Faculty at UIs have already been successful in piloting genomic data science curriculum. The St. Mary's University B.S. in bioinformatics (Supplemental Table S4) was launched in the Fall of 2019 and currently has 43 students enrolled in the program (Fig. 3). The genomics and bioinformatics program at Virginia State University provides training in genomics, computer science, and statistics for students from different majors including biology, computer science, mathematics, and agriculture. These students take the same high-level genomics and programming for bioinformatics courses during their third year and conduct research throughout their degree, culminating in a 1-yr extensive research course during their senior year.

Partnerships among research and undergraduate-focused institutions can be a powerful tool for modeling transferability and curriculum standardization. For example, New Jersey's 3 + 1 program (Rowan College at Burlington County 2021), University of California–Los Angeles’ Center for Community College partnerships (https://www.aap.ucla.edu/units/cccp/), and Virginia's statewide transfer agreement (https://www.vccs.edu/transfer-programs/) partner CCs with four-year research institutions, reducing the cost burden on students seeking genomic data science degrees. Students obtaining an associate degree and earning a minimum grade point average from Virginia's CCs can guarantee admission to any of more than 30 Virginia colleges and universities, including those offering B.S. degrees in bioinformatics or data science. Faculty and administrators involved with these degree programs at R1 institutions can better support incoming students by offering tailored recommendations, such as suggested prerequisites. This translates to a broader diversity of transfer students set up for success.

### Ignite careers through development and research

Data science careers have grown rapidly in the past decade, providing an exciting opportunity as companies are working hard to fill the global talent gap (Rawlings-Goss 2019). However, students might not be well acquainted with the next steps in their professional career beyond academic credit. Mentorship to help students explore and/or identify further career opportunities would help bridge this gap, whether students are applying to summer research programs, transferring to research institutions, exploring graduate school degrees, or entering the workforce. To remove barriers for applying to summer research programs and other positions, R1 and UIs with larger research programs should create application guides and offer workshops, information sessions, recruiting events, and/or alumni panels. Visual tools for mapping opportunities by location, topic, and experience level (i.e., undergraduate or graduate) could also be useful. Next-step opportunities offered by research institutions should provide clear information about stipends and flexibility, if possible (Jensen et al. 2021). Creating new student-focused symposia, even if small in size, is another straightforward way to engage a broad array of students by building confidence and creating a supportive and engaging experience among peers. Funding agencies and research institutions should be incentivized to make introductions and help build broader networks that include industry, nonprofit, and other nonacademic data scientists. Targeted training for different groups can also be an excellent way to help students transition to next steps in their careers. For example, the summer internship for indigenous peoples in genomics (SING, https://sing.igb.illinois.edu/) workshop trains indigenous peoples in the concepts and methods currently used in genomic research while discussing the uses, misuses, and limitations of genomics as a tool for indigenous peoples’ communities. Finally, students could also be encouraged to consider outside accreditation, such as the existing American Statistical Association accreditation (https://www.amstat.org/ASA/Your-Career/Accreditation.aspx) to show their achievements where internal certificates or programs are unavailable.

From the student perspective, identifying outside learning and professional development requires knowing what and where to search ahead of time. This can be remedied by engaging students earlier during secondary education. Although college visits are useful for building interest in postsecondary education in general, a one-day science congress targeted toward high school students hosted by a college or university could create connections between students and practicing scientists. These connections could help students find opportunities and could even evolve to closer mentor–mentee relationships. For students already at two-year or four-year teaching institutions, faculty and students would benefit from attending events like the Annual Biomedical Research Conference for Minority Students (https://abrcms.org) or the Society for Advancement of Chicanos/Hispanics and Native Americans in Science annual meeting (https://www.sacnas.org/), which provide pathways for further experiences and networking.

## Fund the future

Faculty members at UIs have been historically disadvantaged when competing for funding owing to the high teaching load and/or perceived lower research quality, lack of research infrastructure and research personnel, and stigma associated with reanalyzing existing data, among other reasons (Ginther et al. 2011; Hemming et al. 2019). Systemic biases also exist owing to a lack of understanding of the importance of institutions that enroll significant numbers of traditionally underrepresented students. Although several pivotal funding opportunities for UIs have emerged in recent years, the GDSCN constituency identified where funding agencies and philanthropies can prioritize resources. These include funds for faculty networking and knowledge exchange, CURE development, and cloud computing, alongside ongoing efforts to strengthen research support.

Events where faculty can network and develop collaborations require minimal funds but are still a major need among UI faculty (Hemming et al. 2019). Such events have long-lasting benefits, as they facilitate novel research, educational materials, and discovery. Faculty at R1 research institutions can also commit to contacting UI faculty to submit grant proposals collaboratively, share open access teaching resources, and/or invite students to participate in external CUREs and other research experiences. Programs can also be enacted to fund R1 faculty research or teaching time at UIs. Going forward, evaluation of larger NIH proposals could incorporate a broader impacts section akin to those required by NSF, where R1 researchers regularly commit to engaging and collaborating with underrepresented communities. Modest funding could also make a sizable difference for recruiting outside speakers. Speakers coming from underrepresented groups and/or with nontraditional backgrounds can often connect more effectively with students of similar backgrounds and should be encouraged to do so with financial compensation. Grants supporting potential role models could also be expanded by government agencies, for-profit companies, and philanthropies. Postdoctoral and early career fellowships are particularly lacking for underrepresented groups.

As mentioned above, there is a much deeper root for the underrepresentation of certain communities in the field of genomics, which may go back to the scientific education they receive in early K–12 levels, besides socioeconomic and family factors. Therefore, to increase the participation of the currently underrepresented communities in the field of genomics, there needs to be greater investment in science and mathematics at the K–12 grade levels and more activities engaging K–12 students in understanding biology, genomics, and related sciences. Besides improving the overall education quality and particularly science and mathematics education in grade schools, federal funding agencies, and philanthropies should encourage faculty with expertise in genomics working at either UIs or R1 universities to provide training to science and mathematics teachers at high schools, secondary schools, and even primary schools and allow financial support to do so.

The NIH and NSF provide grants for curriculum development, such as the NIH research enhancement award for small-scale research projects at educational institutions (National Institutes of Health 2021d), the advancing innovation and impact in undergraduate STEM education at two-year institutions of higher education program (National Science Foundation 2021), and the historically black colleges and universities–undergraduate program (National Science Foundation 2020). Yet, the percentage of funding granted to UIs remains very low. Releasing teaching time also requires other faculty to shoulder the released teaching load, making it difficult to implement these programs without significant funding. Similarly, the successful NSF REU program (National Science Foundation 2019b) also supports relatively few students, typically only about 10 students per site, which is orders of magnitude smaller than is necessary to give all students an opportunity. Funding programs that sponsor scalable and accessible CUREs can likely provide greater collective impact (Elgin et al. 2021). Philanthropies and private companies could also consider partnering with UI faculty to fund CURE modules in specific topic areas.

More generally, funders must support research in underrepresented communities and at UIs. UIs are typically under much higher economic pressure, making it difficult for faculty to get started with research. First, funders must consider computing costs as part of the solution. Options include streamlining funding for cloud computing through programs like NIH Strides and public-private partnerships like the NSF CISE-MSI program (NSF directorate for computer and information science and engineering with support from Google) (Supplemental Table S2). Importantly, platforms should consider a free no strings–attached tier for users in which compute hours regenerate over time. This prevents accidental overspend, makes it easier to get started, and makes practicing with students simple. Although government agencies have been increasing support for UI research in recent years, companies and private philanthropies can often adopt change more quickly. For example, the Chan Zuckerberg Initiative (CZI) essential open-source software program (https://chanzuckerberg.com/rfa/essential-open-source-software-for-science/) aims to improve reproducibility and transparency in biology and medicine while explicitly seeking applications that increase diversity and inclusion. Biotechnology companies can work directly with UIs to offer support and a path to employment for students. Other research challenges require creative solutions. For example, UIs often lack institutional journal access, which could be overcome with sponsorship by R1 institution libraries. Sponsoring staff positions can also work toward reducing the administrative burden on UI faculty. Funders will need to continue to engage their target communities to provide usable and meaningful support. The GDSCN has created a growing database of resources (https://www.gdscn.org/resources) as a starting point for bringing coursework, development, and funding resources to UIs and their communities.

## The GDSCN vision

We propose a vision in which researchers, educators, and students from diverse backgrounds are able to fully participate in genomic data science research. UIs make education more accessible to students from underrepresented groups. Our strategy is to provide resources that are currently absent at these key institutions to augment the unique strengths that these institutions already provide. As a first step toward this vision, we planted the seeds for a GDSCN by bringing together faculty from CCs, HBCUs, HSIs, and TCUs alongside faculty and staff from Johns Hopkins University and NHGRI. The vision and recommendations in this paper represent the culmination of a year-long process that involved one-on-one virtual meetings and four virtual synchronous events along with several rounds of asynchronous writing sessions.

In addition to bringing faculty together, the pilot efforts of the GDSCN has also supported tangible resources. First, we are developing several modules of genomic data science curricula specifically targeted toward UI student audiences, which are available at https://www.gdscn.org/curricula/courses. Through NHGRI, the GDSCN and other UI faculty members are provided access to cloud computing credits on the AnVIL platform in order to run these courses in their classrooms. Simultaneously, we organized a series of symposiums, in which we crowdsourced challenges, needs, and opportunities in the current academic infrastructure, as well as barriers that need to be overcome to enable broader participation and inclusion in genomic data science research. Finally, we initiated the development of several modules within the Open Case Studies Project (https://www.opencasestudies.org/). In the future, we hope to grow the network (https://www.gdscn.org/contact-us) to incorporate more perspectives and support more faculty from UIs to meet their networking, research, and education goals. We plan to continue iterating on our initiatives via GDSCN faculty surveys. We will likewise record the number of faculty and students reached in training events, classroom time, and upcoming events (such as grant writing workshops).

More broadly, the National Institutes of Health (NIH) has identified four major underrepresented groups whose participation is needed to increase diversity in biomedical research: racial and ethnic groups, people with disabilities, people with low socioeconomic status, and women (National Institutes of Health 2019). In this perspective, we focus on UIs, including MSIs. Our current scope does not specifically include people with disabilities and women. However, both of these are identities that intersect with UIs. We expect that the actions discussed here (Fig. 2), such as greater access to CUREs, exploration of relevant research topics and issues, and expanded funding, will be relevant to other underrepresented communities. However, the GDSCN will need to have direct engagement with women and people with disabilities in the genomic data science community to determine the best way to leverage their specific strengths and advocate for their most pressing needs.

In the future, we hope that faculty at R1 institutions—who identify with an underrepresented group or share an interest in improving the education and research in genomic data science at the institutions serving these students—will consider lending their perspective to the community and reach out for collaborations with the faculty and students at these institutions. Ultimately, we are hopeful that these collaborations will empower scientists to solve key problems, such as the pervasive bias in genomic data collection (Sirugo et al. 2019) and ongoing challenges with accountability, data sovereignty, community/tribal consent, and data misuse (Hudson et al. 2020). Although we have limited our initial efforts to institutions in the United States, we believe that lessons learned among the GDSCN will spark discussions in the global research community. We also believe that GDSCN efforts will be improved by including student and tribal representatives in the conversation (Claw et al. 2021). Finally, we hope that our insights and materials will encourage scientists and their teams to learn and actively support UI communities, ultimately improving access, removing barriers, and making science more innovative and inclusive as a whole.

## The Genomic Data Science Community Network

Rosa Alcazar,^2^ Maria Alvarez,^3^ Rachel Arnold,^4^ Mentewab Ayalew,^5^ Lyle G. Best,^6^ Michael C. Campbell,^7^ Kamal Chowdhury,^8^ Katherine E.L. Cox,^9^ Christina Daulton,^10^ Youping Deng,^11^ Carla Easter,^12^ Karla Fuller,^13^ Shazia Tabassum Hakim,^14^ Ava M. Hoffman,^9^,^15^ Natalie Kucher,^16^ Andrew Lee,^17^ Joslynn Lee,^18^ Jeffrey T. Leek,^9^,^15^ Robert Meller,^19^ Loyda B. Méndez,^20^ Miguel P. Méndez-González,^21^ Stephen Mosher,^16^ Michele Nishiguchi,^22^ Siddharth Pratap,^23^ Tiffany Rolle,^10^ Sourav Roy,^24^ Rachel Saidi,^25^ Michael C. Schatz,^26^ Shurjo K. Sen,^10^ James Sniezek,^27^ Edu Suarez Martinez,^28^ Frederick J. Tan,^29^ Jennifer Vessio,^16^ Karriem Watson,^30^ Wendy Westbroek,^31^ Joseph Wilcox,^32^ Carrie Wright,^9^,^15^ Xianfa Xie,^33^

## Supplementary Material

Supplemental Material

## Footnotes

2 Clovis Community College, Fresno, CA 93730, USA

3 Biology Department, El Paso Community College, El Paso, TX 79924, USA

4 US Fish and Wildlife (Northwest Indian College), Onalaska, WI 54650, USA

5 Biology Department, Spelman College, Atlanta, GA 30314, USA

6 Turtle Mountain Community College, Belcourt, ND 58316, USA

7 Department of Biological Sciences, University of Southern California, Los Angeles, CA 90089, USA

8 Biology Department, Claflin University, Orangeburg, SC 29115, USA

9 Department of Biostatistics, Johns Hopkins Bloomberg School of Public Health, Baltimore, MD 21205, USA

10 National Human Genome Research Institute, National Institutes of Health, Bethesda, MD 20892, USA

11 Department of Quantitative Health Sciences, University of Hawaii at Manoa, Honolulu, HI 96822, USA

12 Smithsonian Institute National Museum of Natural History, Washington, DC 20560, USA

13 Guttman Community College, New York, NY 10018, USA

14 Department of Microbiology and Biomedical Sciences, Dine College, Tuba City, AZ 86045, USA

15 Fred Hutchinson Cancer Research Center, Seattle, WA 98109, USA

16 Department of Biology, Johns Hopkins University, Baltimore, MD 21218, USA

17 Department of Biology, Northern Virginia Community College–Alexandria, Alexandria, VA 22311, USA

18 Department of Chemistry and Biochemistry, Fort Lewis College, Durango, CO 81301, USA

19 Department of Neurobiology, Morehouse School of Medicine, Atlanta, GA 30310, USA

20 Science and Technology, Universidad Ana G. Méndez–Carolina, Carolina, 00983, Puerto Rico

21 Natural Sciences Department, University of Puerto Rico at Aguadilla, Aguadilla, 00603, Puerto Rico

22 Department of Molecular and Cell Biology, University of California, Merced, Merced, CA 95343, USA

23 School of Graduate Studies and Research, Meharry Medical College, Nashville, TN 37208, USA

24 Department of Biological Sciences and Border Biomedical Research Center, University of Texas at El Paso, El Paso, TX 79968, USA

25 Department of Math, Statistics, and Data Science, Montgomery College, Rockville, MD 20850, USA

26 Departments of Biology and Computer Science, Johns Hopkins University, Baltimore, MD 21218, USA

27 Chemical and Biological Sciences, Montgomery College, Germantown, MD 20876, USA

28 Department of Biology, University of Puerto Rico–Ponce, Ponce, 00732, Puerto Rico

29 Department of Embryology, Carnegie Institution, Baltimore, MD 21218, USA

30 National Institutes of Health, Bethesda, MD 20892, USA

31 Department of Biology, Flathead Valley Community College, Kalispell, MT 59901, USA

32 Department of Biology, Nevada State College, Henderson, NV 89002, USA

33 Department of Biology, Virginia State University, Petersburg, VA 23806, USA
