# Supplementary material for: Diversifying the genomic data science research community
Source: Genome Res. 2022 Jul;32(7):1231–41. doi: 10.1101/gr.276496.121 (PMC9341509; doi:10.1101/gr.276496.121)
Supplement: Supplemental Material [file supp_32_7_1231__DC1.html]

Diversifying the genomic data science research community — Diversifying the genomic data science research community — Supplemental Material 

# Diversifying the genomic data science research community

## Supplemental Material

- Supplemental\_Material.pdf
