# Supplementary material for: Diversifying the genomic data science research community
Source: Genome Res. 2022 Jul;32(7):1231–41. doi: 10.1101/gr.276496.121 (PMC9341509; doi:10.1101/gr.276496.121)
Supplement: Supplemental Material [file supp_gr.276496.121_Supplemental_Material.pdf]

**Supplemental information to accompany:**

*Diversifying the Genomic Data Science Research Community*

**Text S1:** Faculty and staff at UIs (Underserved Institutions) possess unique strengths and have experience with the specific interests, needs, challenges, and concerns of the populations they serve. We briefly describe these unique attributes below.

- ***Community Colleges (CCs)*** have smaller class sizes at the freshman and sophomore levels and provide unique student-centered support (Holmberg et al. 2021). Tuition at CCs is extremely affordable compared to public universities (on average only one third the cost), and flexible class schedules accommodate working students. CCs tend to be more agile in responding to the needs of the community and provide support to traditional and non-traditional students alike, often supplying more personalized attention. CCs promote a culture of inclusion and innovation while making higher education more accessible to individuals across socioeconomic groups. Large teaching loads (e.g., 4 lectures and 3 labs per semester) with no release time are typical for CC faculty. Research laboratories, equipment, and funds for laboratory supplies are limited or completely absent, making experimental work extremely challenging, but data science offers an exciting opportunity.
- ***Historically Black Colleges and Universities (HBCUs)*** are incredibly diverse and include institutions that focus on liberal arts, business, professional degrees, workforce development, or cutting-edge research. HBCUs have been key to increasing participation and success of minority students in STEM (Gasman and Nguyen 2016) and provide an affordable, supportive learning environment for minority student achievement (Harper 2019). HBCUs serve as pillars of Black and African American history, art, culture, and politics, attracting an extremely diverse student body and faculty community. However, the legacy of slavery, segregation, and systemic social discrimination means that HBCUs tend to have financial challenges (Harper 2019). Some faculty members at HBCUs are developing new federally funded education and research programs to provide students training in genomics and data sciences. However, the long-term success of these programs requires wider acceptance by HBCU faculty colleagues and support from administrations in the form of teaching release time, compensation, technical personnel, and adequate genomics and data science research facilities.

- ***Hispanic-Serving Institutions (HSIs)*** provide an essential resource making education accessible to the nation’s growing population of Hispanic Americans. HSIs enroll two-thirds of all Hispanic undergraduates and are among the top institutions with respect to the Social Mobility Index (SMI) (Hispanic Association of Colleges and Universities 2021). Students choose HSIs for their exceptional affordability and proximity to home and family (Cuellar 2019). Like HBCUs, HSIs are under-resourced. These diverse institutions receive only 68 cents for every federal funding dollar granted to all other institutions per student annually (Hispanic Association of Colleges and Universities 2021)).
- ***Tribal Colleges and Universities (TCUs)*** provide culturally relevant, geographically accessible, and affordable higher education. Indigenous ways of thinking bring diverse perspectives and solutions to health and environmental questions. TCUs are centered around the communities they serve, forming bridges to meaningful community engagement. Outside collaborations with TCUs must be flexible, reciprocal, and culturally sensitive. TCUs and affiliated tribal communities require engagement through every step of the research and data sharing process (Hudson et al. 2020). Indigenous scientists are wary of “helicopter research”; collaborations should create meaningful educational and training materials that can lead to co-publication and co-presentation (Guglielmi 2019; Fox 2020). Activities should be sustainable and empowering for TCU faculty. Academic researchers engaged in tribal projects should become familiar with tribal sovereignty and ethics and informed consent (Garrison et al. 2019) as well as community education around genetics (Claw et al. 2021). Collaborative research projects and training opportunities may focus on tribal health or environmental priorities. TCUs are continuing to work on their technology and internet infrastructure to increase access in remote areas.

**Table S1:** Organizations supporting the needs of underserved subpopulations have emerged in recent years. These groups are vital to developing a sense of belonging and support system. Some examples of these affinity groups include those listed here.

| Organization | Website |
|---|---|
| Center for First-generation Student Success | https://firstgen.naspa.org/ |
| Student Veterans Research Network | https://www.svrn.org/ |
| Society for Advancement of Chicanos/Hispanics and Native Americans in Science | https://www.sacnas.org/ |
| American Indian Science and Engineering Society | https://www.aises.org/ |
| Native BioData Consortium | https://indigidata.nativebio.org/ |
| Annual Biomedical Research Conference for Minority Students | https://abrcms.org/ |
| National Foster Youth Institute | https://nfyi.org/issues/higher-education/ |
| From Prison Cells to PhD | https://www.fromprisoncellstophd.org/ |

**Table S2**: Cloud-based computing resources. Note that other tools, such as JupyterHub (https://jupyter.org/hub#deploy-a-jupyterhub) and Binder (https://mybinder.org/), can also make it easier to deploy notebooks for multiple users on commercially available clouds using Kubernetes. The Galaxy Server Directory (https://galaxyproject.org/use/) also includes a comprehensive list of more cloud providers providing access to Galaxy. Combinations of open source software (e.g., Docker and R, https://www.docker.com/blog/docker-higher-education-tools-resources-teachers/) can also provide an alternative for modular teaching needs. Online resources change frequently; this information was collected on Dec 7, 2021.

| Resource | Interface | Costs | Strengths / Weaknesses |
|---|---|---|---|
| | | ***Project Specific*** | |
| AnVIL / Terra https://anvilproject.org/learn | Terra portal for launching cloud environments with workflows, Jupyter Notebook, RStudio, Galaxy, and Command Line Interface; hosts open and controlled NHGRI datasets | **Compute** starting at $0.06 per hr; possible to obtain $300 in credits through Google Cloud **Persistent disk** $4.00 per month per 100GB | **Strengths:** Collaborative work on sensitive/protected and NHGRI datasets, many options for different experience levels, extremely scalable **Weaknesses:** Lacks a free tier for beginners |
| All of Us https://www.researchallofus.org/ | Terra portal that includes analysis workspaces and Jupyter Notebook; hosts open and protected tier for All of Us Research Program participant data | **Compute** starting at $0.06 per hr; possible to obtain $300 in credits through Google Cloud **Persistent disk** $4.00 per month per 100GB | **Strengths:** Access and ability to build datasets, built in analysis spaces **Weaknesses:** Lacks a free tier for beginners |
| BioData Catalyst https://biodatacatalyst.nhlbi.nih.gov | Terra and Seven Bridges portal that includes analysis workflows and RStudio and Jupyter Notebook; hosts protected NHLBI datasets | **Compute** costs vary depending on AWS instance used; possible to obtain $500 in credits **Persistent disk** $2.10 per month per 100GB through Seven Bridges | **Strengths:** Access to datasets, built in workflows/pipelines **Weaknesses:** Lacks a free tier for beginners |
| Cancer Research Data Commons https://datacommons.cancer.gov/ | Terra, Seven Bridges, and ISB-CGC portal that includes analysis workflows, genomic tools, RStudio, and Jupyter Notebook; hosts open and controlled datasets from NCI programs and key external cancer programs | **Compute** and **Persistent disk** costs vary depending on platform used, possible to obtain $300 in credits through Google Cloud | **Strengths:** Access to datasets, built in workflows/pipelines, flexible with many options for different experience levels **Weaknesses:** Lacks a free tier for beginners |
| Kids First DRC https://kidsfirstdrc.org | Storage, sharing, and analysis portal supported by Cavatica / Seven Bridges with workflows and genomics apps; hosts open and controlled datasets | **Compute** costs vary depending on AWS instance used; possible to obtain $500 in credits **Persistent disk** $2.10 per month per 100GB through Seven Bridges | **Strengths:** Access to datasets, built in workflows/pipelines **Weaknesses:** Lacks a free tier for beginners, less flexibility |
| UK Biobank https://ukbiobank.dnanexus.com/landing | DNANexus portal for launching cloud environments with workflows, JupyterLab, Command | **Compute** and **Persistent disk** costs vary depending on AWS instance used; users start with | **Strengths:** Access to datasets, flexible with many options for analysis |

| | | | |
|---|---|---|---|
| | Line Interface, or custom tools; hosts the UK Biobank dataset | approximately $50 in free compute and storage credit | **Weaknesses:** Learning curve might be steeper compared to other platforms |
| | ***Academic*** | | |
| CyVerse https://learning.cyverse.org/ | Launching platform for maintained images, including those with bioInformatics tools, Jupyter Notebook, RStudio, and Galaxy | **Compute** free with short/moderate wait times with subscriptions available **Persistent disk** free up to 100GB, starting at $100 per TB per year for >100GB. | **Strengths:** Affordable, many options for different experience levels **Weaknesses:** Requires permission to access resources |
| Galaxy https://training.galaxyproject.org/ | Point-and-click for commonly used genomics tools | **Compute** free tier limited to 6 compute jobs for registered users, 1 for unregistered users (short/moderate wait times) **Persistent disk** free tier limited to 250GB for registered users, 5GB for unregistered users | **Strengths:** No programming experience needed, powerful workflows, free for basic analyses **Weaknesses:** Wait times/limits, limited to tools available |
| GenePattern https://www.genepattern.org/user-guide (Reich et al. 2006) | Point-and-click for commonly used genomics tools, plus Jupyter Notebook | **Compute** free tier with short/moderate wait times **Persistent disk** free public server limited to 30GB. | **Strengths:** No programming experience needed, free for basic analyses **Weaknesses:** limited to tools available, less active user community |
| Jetstream https://jetstream-cloud.org/documentation-training/index.html (Stewart et al. 2015) | Launching platform for maintained images, including those with bioInformatics tools, R, Galaxy, and more | **Compute** free tier up to 50,000 Virtual CPU Hours **Persistent disk** allocations provided on a per-project basis; Allocation requests for education and research available (https://docs.jetstream-cloud.org/faq/alloc/). | **Strengths:** Affordable, many options for different experience levels **Weaknesses:** Requires permission to access resources |
| KBase https://www.kbase.us/learn/ (Arkin et al. 2018) | Point-and-click for commonly used genomics tools, plus Jupyter Notebook | **Compute** free tier with short/moderate wait times. **Persistent disk** has no strict storage limits | **Strengths:** Easy start on free tier, No programming experience needed **Weaknesses:** Less flexible, limited to tools available |
| SciServer https://www.sciserver.org/support/ (Taghizadeh-Popp et al. 2020) | Launching platform for maintained images, including those with bioInformatics tools, R, python, and more; hosts datasets | **Compute** free tier with short/moderate wait times **Persistent disk** free up to 10GB permanent storage; temporary storage at 1TB+ | **Strengths:** Access to large datasets, flexible with many options for analysis; great option for collaborating with physicists and other multidisciplinary team members **Weaknesses:** Learning curve might be steeper compared to other platforms |
| | ***Commercial*** | | |
| Illumina BaseSpace https://basespace.illumina.com/ | Point-and-click for commonly used genomics tools | **Compute** with limited free basic tier; credits can be purchased **Persistent disk** free tier limited to 1TB; credits can be purchased | **Strengths:** Built-in workflows, collaborative work on sensitive/protected datasets **Weaknesses:** Less flexible, more expensive than others |
| Google Colaboratory https://colab.research.google.com/ | Jupyter notebook (Python) | **Compute** free tier resources are limited, usage limits fluctuate **Persistent disk** free up to 5GB | **Strengths:** Easy start on free tier, Quickly code live with other users |

| | | | |
|---|---|---|---|
| | | | **Weaknesses:** Connection and RAM not guaranteed, limited languages/tools |
| RStudio Cloud<br>https://rstudio.cloud/ | RStudio accessed through browser | **Compute** free tier up to 25 hours, paid tier $0.10 per hour<br>**Persistent disk** free tier limited to 20GB per project | **Strengths:** Easy start on free tier<br>**Weaknesses:** limited to R based languages/tools |
| Seven Bridges<br>https://www.sevenbridges.com/platform/ | Point-and-click for commonly used genomics tools, RStudio, command line interface | **Compute** and **Persistent disk** prices determined by AWS negotiated price | **Strengths:** Flexible and scalable, with built in genomics tools, relatively affordable<br>**Weaknesses:** No free tier for beginners |

**Table S3:** Learning resources to supplement courses in genomic data science. MOOC: Massive online open course. If needed, subscription or paid services should be covered by funding sources.

| Description | Resource |
|---|---|
| Standalone MOOC / Course for genomic data science | • Coursera Genomic Data Science Specialization (https://www.coursera.org/specializations/genomic-data-science)<br>• Bioinformatics Algorithms (https://www.bioinformaticsalgorithms.org/) |
| Project based learning modules (open source) | • Open Case Studies Project (https://www.opencasestudies.org/) |
| Lesson planning network; modules focused on bioinformatics and data science (open source) | • Quantitative Undergraduate Biology Education and Synthesis Hub (QUBES, https://qubeshub.org/publications/browse) (Donovan et al. 2015) |
| Modular training for genetics, genomics, and bioinformatics (open source) | • CyVerse tutorials (https://learning.cyverse.org/en/latest/tutorials.html)<br>• Galaxy Training Network (https://training.galaxyproject.org/)<br>• Orchestra (http://app.orchestra.cancerdatasci.org/)<br>• Babraham Institute bioinformatics courses (https://www.bioinformatics.babraham.ac.uk/training.html)<br>• XBio Cell Biology & Genetics (https://explorebiology.org/collections/genetics) |
| Modular training for data science (open source) | • Tidyverse Skills for Data Science in R (https://leanpub.com/tidyverseskillsdatascience)<br>• Learn-R (https://www.learn-r.org/)<br>• Swirl (https://swirlstats.com/)<br>• R for Data Science (https://r4ds.had.co.nz/)<br>• Data Science in Practice (https://datascienceinpractice.github.io/) (Donoghue, Voytek, and Ellis 2021)<br>• The Carpentries (https://carpentries.org/)<br>• SciServer Courseware (https://www.sciserver.org/outreach/)<br>• Python for Biologists (https://www.pythonforbiologists.org/) |
| Modular training (fee- or subscription-based) | • DataQuest (https://www.dataquest.io/)<br>• Codecademy (https://www.codecademy.com)<br>• Data Camp (https://www.datacamp.com/)<br>• SimBio (https://simbio.com/) |
| Modular training for K-12 | • DataNuggets (http://datanuggets.org/) (Schultheis and Kjelvik 2015)<br>• DataSpire (https://dataspire.org)<br>• Oak Ridge Institute for Science and Education (https://orise.orau.gov/resources/k12/lesson-plans.html)<br>• YouCubed (https://www.youcubed.org/) |

**Table S4:** List of courses and topics which could be included in an accredited degree program, based on the St. Mary's University B.S. in Bioinformatics. Prerequisites include General Biology for Majors, General Chemistry, and Calculus I; corequisites include Fundamentals of programming/software development and General Physics. For more information, see https://catalog.stmarytx.edu/undergraduate/majors-programs/science-engineering-technology/bioinformatics/bioinformatics-bl/#degreeplantext

| Course name | Topics | Learning Objectives |
|---|---|---|
| Introduction to Bioinformatics (w/lab) | Basics of genomics and bioinformatics<br>Human genome and browser<br>Pairwise Sequence alignment<br>Multiple Sequence alignment<br>Sequence Database Search<br>Sequence polymorphism<br>Phylogenetic analysis<br>Gene finding/prediction<br>RNA sequences: prediction and analysis of structures | The objective of this course is to teach how computational techniques can help with solving biological problems. Students will learn to efficiently use multiple genomics and bioinformatics tools, that are freely available, for the analysis of DNA, RNA and protein sequences and structure. No programming skills are necessary for this course. |
| Biostatistics for Life Sciences | How to use R for data analysis.<br>Samples and Populations<br>Linear Regression<br>Comparison of Groups<br>The Normal Distribution<br>Statistical Models, Estimation, and Confidence Intervals<br>Hypothesis Tests<br>Probabilities<br>The Binomial Distribution<br>Logistic Regression<br>Survival Analysis | This course will provide the background and application of statistical tools for analyzing different types of data frequently encountered by life scientists. The emphasis will be on the applications of various statistical methodologies on biological data, using the R programming language. |
| Genes, Genomes, and Genomics (w/lab) | From Genes to Genomes: basic molecular biology<br>How to clone a gene<br>Genomic and cDNA Libraries<br>Polymerase Chain Reaction<br>Sequencing a Cloned Gene<br>Analysis of Gene Expression<br>Products from Native and Manipulated Cloned Genes<br>Genomic Analysis<br>Analysis of Genetic Variation<br>Transgenesis | The objective of this course is to teach students the basics of molecular biology. The focus of this course will be on genes, genomes and genomics. This will include strategies to clone a gene and prepare genomic and cDNA libraries. Students will learn the basics of DNA sequencing. They will also learn to analyze gene expression. The course will also focus on analysis of genomes and genetic variation. Students will also learn about transgenesis. |

| | | |
|---|---|---|
| Transcriptomics, Proteomics, and Metabolomics (w/lab) | DNA microarray technology<br>Challenges and Future Trends in DNA Microarray Analysis<br>Next Generation Sequencing: New Tools<br>Overview of Quantitative Proteomic Approaches<br>Overview of Protein Microarrays<br>Transcriptome and Metabolome Data Integration<br>Identification of Biomarkers and Biochemical Pathway Visualization<br>Functional Glycomics Analysis: Challenges and Methodologies<br>Applications of Glycan Microarrays to Functional Glycomics<br>Bioinformatic Analysis of Gene Expression Data<br>Transcriptome and Metabolome Data Integration | The objective of this course is to teach students the fundamental aspects of the new instrumental and methodological developments in omics technologies, including those related to genomics, transcriptomics, epigenetics, proteomics and metabolomics. The focus of this course will be on DNA microarray analysis, next-generation sequencing technologies, genome-wide analysis of methylation and histone modifications. Students will learn emerging techniques in proteomics and recent quantitative proteomics approaches. They will also learn the basics of metabolomics and metabolome analysis. The course will also focus on statistical approaches for the analysis of microarray data, the integration of transcriptome and metabolome data and computational approaches for visualization and integration of omics data. |
| Algorithms for Computational Biology with PERL/Python | Linux for bioinformatics<br>Getting started with Perl/Python<br>Individual approaches to programming<br>Representing and Manipulating sequence data<br>Using PERL/Python documentation<br>Motifs and Loops<br>Operating Strings and Arrays<br>Regular expressions<br>Hashes and Data Structures<br>Creating Subroutines | The objective of this course is to teach students the basics of theLinux environment and PERL scripting. Students will learn how to write PERL scripts for solving biological problems. The focus of this course will be on designing algorithms to manipulate and analyze sequence data. This will include programming strategies to store and concatenate DNA sequences. Writing scripts to generate complementary and reverse complementary sequences. Students will learn how to work with files and arrays. They will also learn to generate random numbers and simulate DNA mutations. The course will also focus on hashes and data structures. |
| Programming for Bioinformatics with R | DNA Sequence Statistics<br>Sequence Databases<br>Pairwise Sequence Alignment<br>Multiple Alignment and Phylogenetic trees<br>Computational Gene-finding<br>Comparative Genomics<br>Hidden Markov Models<br>Protein-Protein Interaction Graphs | The objective of this course is to teach students bioinformatics programming techniques using R. The focus of this course will be on development of programs to perform commonly used bioinformatics techniques like pairwise and multiple sequence alignments. Students will learn computational gene-finding and comparative genomic techniques. They will also learn to create phylogenetic trees and protein-protein interaction graphs. Students will also learn about Hidden Markov models. |

| | | |
|---|---|---|
| Big Data Concepts | Basic Big Data Concepts<br>Linux for Big Data Analysis<br>Python for Big Data Analysis<br>R for Big Data Analysis<br>Genome-Seq Data Analysis<br>RNA-Seq Data Analysis<br>Microbiome-Seq Data Analysis<br>miRNA-Seq Data Analysis<br>ChIP-Seq Data Analysis<br>Big Data and Drug Discovery | The objective of this course is to teach students the basic big data concepts. The focus of this course will be on big data analysis. Students will learn emerging techniques in proteomics and recent quantitative proteomics approaches. They will also learn the basics of the platforms and programming languages used for big data analysis. The course will also focus on the analysis of Genome-, RNA-, miRNA-, Microbiome- and ChIP-sequencing data. Students will also learn about the usage of big data in drug discovery. |
| Bioinformatics Internship | 120 hrs of documented internship or research. | The objective of this course is to provide an opportunity for students in Bioinformatics major to participate in real-life bioinformatics internship or research. This course will be for seniors and juniors. Emphasis will be placed on commonly used genomics/transcriptomics/proteomics/metabolomics projects and the use of standard operating laboratory/industry procedures. Examples of potential collaborative organizations include medical/health centers, molecular genomics labs/companies, computational biology labs/companies, software development/labs companies and biostatistics labs/companies. |
| Bioinformatics Capstone | **Thesis -**<br>Prior to the end of the semester, write and submit a 25-30 page, double-spaced summary of the research to the capstone instructor. This will include an abstract, introduction, materials and methods, results and discussion and conclusion and references.<br>The thesis should have at least 3 figures with legends and descriptions.<br>**Oral presentation**<br>A 20 minutes' powerpoint presentation consisting of a minimum of 20 slides<br>Oral presentation will be on the same research that is presented in the thesis<br>5 minutes of Question and Answer sessions with peers and faculty members | The objective of this integrative Capstone is to provide students with an opportunity to write a thesis on their research and present it in the form of an oral presentation. This course will promote advanced scientific writing and broad perspectives of issues in current Bioinformatics research. Students will demonstrate their ability to integrate concepts to a practical situation by presenting a thesis on the research they have performed in an industrial or academic setting. The capstone must be taken during the senior year. |

## References


Arkin, Adam P., Robert W. Cottingham, Christopher S. Henry, Nomi L. Harris, Rick L. Stevens, Sergei Maslov, Paramvir Dehal, et al. 2018. “KBase: The United States Department of Energy Systems Biology Knowledgebase.” *Nature Biotechnology* 36 (7): 566–69.

Claw, Katrina G., Nicolas Dundas, Michael S. Parrish, Rene L. Begay, Travis L. Teller, Nanibaa’ A. Garrison, and Franklin Sage. 2021. “Perspectives on Genetic Research: Results From a Survey of Navajo Community Members.” *Frontiers in Genetics* 12 (December): 734529.

Cuellar, Marcela G. 2019. “Creating Hispanic-Serving Institutions (HSIs) and Emerging HSIs: Latina/o College Choice at 4-Year Institutions.” *American Journal of Education* 125 (2): 231–58.

Donoghue, Thomas, Bradley Voytek, and Shannon E. Ellis. 2021. “Teaching Creative and Practical Data Science at Scale.” *Journal of Statistics and Data Science Education* 29 (sup1): S27–39.

Donovan, Sam, Carrie Diaz Eaton, Stith T. Gower, Kristin P. Jenkins, M. Drew LaMar, Dorothybelle Poli, Robert Sheehy, and Jeremy M. Wojdak. 2015. “QUBES: A Community Focused on Supporting Teaching and Learning in Quantitative Biology.” *Letters in Biomathematics* 2 (1): 46–55.

Fox, Keolu. 2020. “The Illusion of Inclusion - The ‘All of Us’ Research Program and Indigenous Peoples’ DNA.” *The New England Journal of Medicine* 383 (5): 411–13.

Garrison, Nanibaa’ A., Māui Hudson, Leah L. Ballantyne, Ibrahim Garba, Andrew Martinez, Maile Taualii, Laura Arbour, Nadine R. Caron, and Stephanie Carroll Rainie. 2019. “Genomic Research Through an Indigenous Lens: Understanding the Expectations.” *Annual Review of Genomics and Human Genetics* 20 (August): 495–517.

Gasman, Marybeth, and Thai-Huy Nguyen. 2016. “Engaging Voices: Methods for Studying STEM Education at Historically Black Colleges and Universities (HBCUs).” *Journal for Multicultural Education* 10 (2): 194–205.

Guglielmi, Giorgia. 2019. “Facing up to Injustice in Genome Science.” *Nature* 568 (7752): 290–93.

Harper, Brian E. 2019. “African American Access to Higher Education: The Evolving Role of Historically Black Colleges and Universities.” *American Academic* 3 (January). http://hdl.handle.net/10919/86971.

Hispanic Association of Colleges and Universities. 2021. “2021 Hispanic Higher Education and HSIs Facts.” Hispanic Association of Colleges and Universities. April 6, 2021. https://www.hacu.net/hacu/HSI_Fact_Sheet.asp.

Holmberg, Tara Jo, Sharon Gusky, Stacey Kiser, Vedham Karpakakunjaram, Heather Seitz, Linnea Fletcher, Lindsey Fields, Apryl Nenortas, Andrew Corless, and Katrina Marcos. 2021. “Biology Educators, Professional Societies, and Practitioner Networks within Community Colleges.” *New Directions for Community Colleges* 2021 (194): 15–28.

Hudson, Maui, Nanibaa’ A. Garrison, Rogena Sterling, Nadine R. Caron, Keolu Fox, Joseph Yracheta, Jane Anderson, et al. 2020. “Rights, Interests and Expectations: Indigenous Perspectives on Unrestricted Access to Genomic Data.” *Nature Reviews. Genetics* 21 (6): 377–84.

Reich, Michael, Ted Liefeld, Joshua Gould, Jim Lerner, Pablo Tamayo, and Jill P. Mesirov. 2006. “GenePattern 2.0.” *Nature Genetics* 38 (5): 500–501.

Schultheis, Elizabeth H., and Melissa K. Kjelvik. 2015. “Data Nuggets.” *The American Biology Teacher* 77 (1): 19–29.

Stewart, Craig A., George Turner, Matthew Vaughn, Niall I. Gaffney, Timothy M. Cockerill, Ian Foster, David Hancock, et al. 2015. “Jetstream.” In *Proceedings of the 2015 XSEDE Conference on Scientific Advancements Enabled by Enhanced Cyberinfrastructure - XSEDE ’15*. New York, New York, USA: ACM Press. https://doi.org/10.1145/2792745.2792774.

Taghizadeh-Popp, M., J. W. Kim, G. Lemson, D. Medvedev, M. J. Raddick, A. S. Szalay, A. R. Thakar, et al. 2020. “SciServer: A Science Platform for Astronomy and beyond.” *Astronomy and Computing* 33 (100412): 100412.
